# A unique bZIP transcription factor imparting multiple stress tolerance in Rice

**DOI:** 10.1186/s12284-019-0316-8

**Published:** 2019-08-02

**Authors:** Priyanka Das, Nita Lakra, Kamlesh Kant Nutan, Sneh Lata Singla-Pareek, Ashwani Pareek

**Affiliations:** 1 Stress Physiology and Molecular Biology Laboratory, School of Life Sciences, Jawaharlal Nehru University, New Delhi, 110067 India; 2 Plant Stress Biology, International Centre for Genetic Engineering and Biotechnology, Aruna Asaf Ali Road, New Delhi, 110067 India

**Keywords:** ABA, bZIP transcription factor, Callose, *Oryza sativa*, Photosynthesis, Salinity, Heat

## Abstract

**Background:**

Rice productivity is adversely affected by environmental stresses. Transcription factors (TFs), as the regulators of gene expression, are the key players contributing to stress tolerance and crop yield. Histone gene binding protein-1b (OsHBP1b) is a TF localized within the *Saltol* QTL in rice. Recently, we have reported the characterization of OsHBP1b in relation to salinity and drought tolerance in a model system tobacco. In the present study, we over-express the full-length gene encoding OsHBP1b in the homologous system (rice) to assess its contribution towards multiple stress tolerance and grain yield.

**Results:**

We provide evidence to show that transgenic rice plants over-expressing *OsHBP1b* exhibit better survival and favourable osmotic parameters under salinity stress than the wild type counterparts. These transgenic plants restricted reactive oxygen species accumulation by exhibiting high antioxidant enzyme activity (ascorbate peroxidase and superoxide dismutase), under salinity conditions. Additionally, these transgenic plants maintained the chlorophyll concentration, organellar structure, photosynthesis and expression of photosynthesis and stress-related genes even when subjected to salinity stress. Experiments conducted for other abiotic stresses such as drought and high temperature revealed improved tolerance in these transgenic plants with better root and shoot growth, better photosynthetic parameters, and enhanced antioxidant enzyme activity, in comparison with WT. Further, the roots of transgenic lines showed large cortical cells and accumulated a good amount of callose, unlike the WT roots, thus enabling them to penetrate hard soil and prevent the entry of harmful ions in the cell.

**Conclusion:**

Collectively, our results show that rice *HBP1b* gene contributes to multiple abiotic stress tolerance through several molecular and physiological pathways and hence, may serve as an important gene for providing multiple stress tolerance and improving crop yield in rice.

**Electronic supplementary material:**

The online version of this article (10.1186/s12284-019-0316-8) contains supplementary material, which is available to authorized users.

## Background

Soil salinity, soil dehydration, and extreme environmental temperatures significantly influence plant growth and development and finally result in compromised crop yield (Cramer et al. 2011). The molecular response of plants towards these stresses has been reported to be highly complex; an interplay of many genes involved in perception and signal transduction (Pareek et al. 2010). Among these genes, the transcription factors (TFs) have been reported as key players in determining the survival and yield of plants under stress conditions (Lakra et al. 2015; Nutan et al. 2017). bZIP (basic leucine zipper) group is one of the major group of transcription factors which has expanded and evolved in genomes to play a crucial role in plant growth and stress response. bZIP proteins are a class of unique TFs with the bZIP domain and two structural features: a leucine zipper domain and a basic DNA binding domain (Nijhawan et al. 2008). bZIP proteins are evidently seen throughout the plant kingdom participating in various physiological processes such as seed germination, flower development and fertility, plant senescence, abiotic stress responses and ABA signal transduction (Lee et al. 2006; Nijhawan et al. 2008; Zou et al. 2008; Alves et al. 2013). Although, transgenic plants over-expressing the bZIP proteins have been reported to exhibit a higher tolerance to abiotic stresses such as salinity, drought and extreme temperature conditions (Lee et al. 2006; Zhang et al. 2008; Liao et al. 2008; Liu et al. 2014; Lakra et al. 2015), the physiological and molecular basis of tolerance in these plants largely remains unexplored. Furthermore, the role of bZIP proteins towards maintaining the yield in crop plants under stress conditions is yet to be reported.

Rice (*Oryza sativa* L.) is a major crop plant and staple food for half of the world’s population (Pareek et al. 2010). But, the fact is that most of the high yielding and popular varieties of rice such as IR64 are sensitive to abiotic stresses. Hence, there is an urgent need to understand the response of this crop towards stresses, especially salinity and drought which are severely affecting the crop yield (Pareek et al. 2010). Through gene expression analysis in salinity tolerant genotypes of rice, it has been clearly established that array of genes are constitutively expressed at a higher level to make the plant salinity tolerant (Kumari et al. 2009; Nutan et al. 2017; Soda et al. 2013). Salinity stress has been documented to induce a set of *Saltol* QTL-localized genes including Histone gene binding protein/transcription factor-1b (HBP1b) which is also differentially expressed among the contrasting genotypes of rice (Kumari et al. 2009).

HBP1b is categorized under bZIP family. According to the structure and DNA binding specificity of the molecule, HBP TFs are divided into two major groups named as; *HBP1a* and *HBP1b* (Jakoby et al. 2002). *HBP1a* are also known as GBFs (G-box binding factors) and are well characterized as they preferentially bind to ‘CACGTG’ and ‘ACGTCA’ sequences. The other conserved group is composed of *HBP1b* which bind to TGACG (T/C) sequences and have a bZIP domain close to the N-terminus. Tabata et al. (1991) have shown the binding of HBP1b TFs to the histone (H3) gene promoter and many reports have revealed that the plant histone genes may play key roles in diverse stress responses (Tabata et al. 1991; Schultz et al. 1996; Bray et al. 1999). As it is clear that the transcription factor HBP1b in rice is salinity inducible (Kumari et al. 2009) and is localized within a QTL for salinity tolerance, we hypothesized that this gene could function as a key player for salinity tolerance in the rice plant. Ectopic expression of this *HBP1b* gene in a heterologous system (tobacco) and analysis of stress tolerance by our group (Lakra et al. 2015) provided the clue that over-expression of this gene could affect the expression of several salinity responsive downstream genes. Additionally, it could also serve as a ‘master gene’ contributing to the improved growth and yield under abiotic stress conditions. To test this theory, we have now over-expressed the full-length HBP1b (Gene ID- LOC_Os01g17260) in the homologous system (*O. sativa* L. cv. IR64) and analysed the behaviour of the transgenic plants over-expressing rice HBP1b under different abiotic stress conditions, with special emphasis on the plant growth and yield under salinity. In the present work, we also dissect out molecular mechanism of stress tolerance in these plants as mediated through the altered expression of the key genes involved in photosynthesis and stress-response.

## Results

### Nuclear-localized bZIP transcription factor *OsHBP1b* exhibits developmental regulation and differential expression under diverse abiotic stresses

**Fig. 1:**
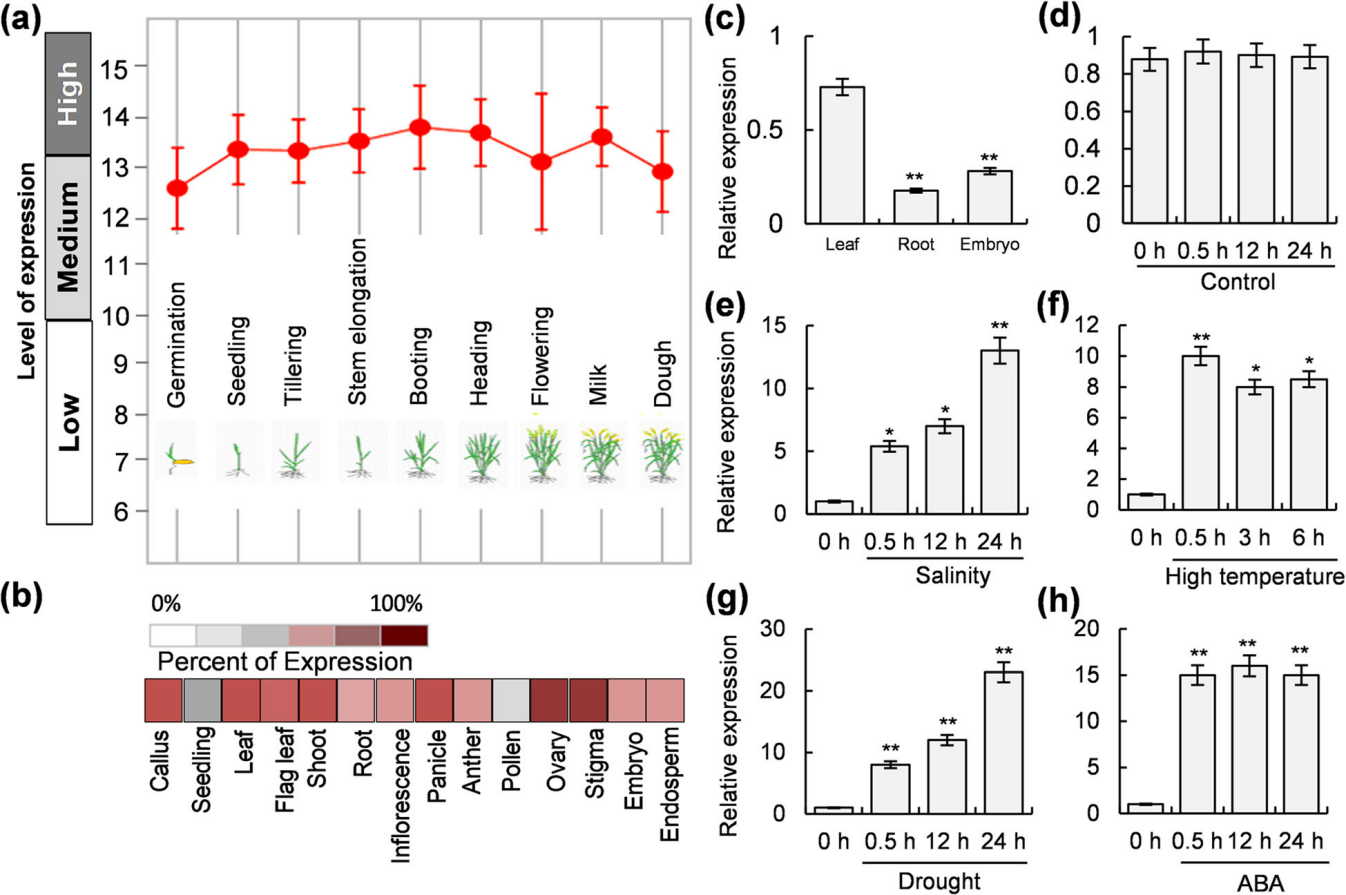
Transcript expression pattern for *OsHBP1b* during different stages of development, in different plant tissues and under different stress conditions in rice. **a** Transcript expression pattern of *OsHBP1b* at different stages of development during the lifecycle of rice based on publicly available microarray data (www.genevestigator.com). **b** Expression pattern of *OsHBP1b* was analysed in different tissue of rice plant based on publicly available microarray data (www.genevestigator.com). **c** Tissue specific expression analysis of *OsHBP1b* in rice was analyzed through quantitative real-time PCR. Expression pattern for OsHBP1b was analyzed under control (**d**) and different abiotic stress (**e**; salinity, **f**; high temperature, **g**; drought, **h**; ABA) treatments with increase in duration of stress period. Expression of eEF2 (housekeeping gene) was taken as control. Error bars show standard error from triplicate experiments. Single asterisk show significant difference at *p* < 0.005 and double asterisk at *p* < 0.001

In our previous study, we have shown that OsHBP1b is a bZIP type protein and is very much related to its orthologs from wheat and barley (Lakra et al. 2015). By GFP-fusion assay, it has also been confirmed that the OsHBP1b protein is localized in the nucleus (Lakra et al. 2015). It has also been reported that transcription factors express at specific stages of plant development to perform a specific role (Ramachandran et al. 1994). These observations prompted us to analyse the transcript abundance for the *OsHBP1b* at different developmental stages of rice. For this purpose, expression levels of *OsHBP1b* were analyzed in rice under different developmental stages through the publicly available microarray databases (https://www.genevestigator.com/gv/). Expression of *OsHBP1b* was checked at nine different growth stages of rice and the results are presented in Fig. 1. The analysis confirmed that *OsHBP1b* transcript is highly abundant in rice during all the developmental stages with its highest expression at the booting stage and lowest expression during the germination stage (Fig. 1a). Furthermore, *OsHBP1b* showed differential transcript abundance in various tissues, where, a medium to high expression was found in different tissue types (Fig. 1b). In comparison to other tissues, pollen tissue showed the lowest expression of *OsHBP1b* transcript (Fig. 1b). In confirmation with the microarray results (Fig. 1b), quantitative real- time PCR analysis showed a higher transcript expression for *OsHBP1b* in the leaf tissue followed by the root and the embryo (Fig. 1c).

In our previous study, we have shown that *OsHBP1b* is localized within the *Saltol* QTL and also inducible by salinity (Lakra et al. 2015). In order to check if this gene is responsive to diverse abiotic stresses in addition to salinity, we have performed expression analysis of *OsHBP1b* under various stress conditions (Fig. 1e - h). We found that though there are no significant changes in the transcript abundance of this gene in rice seedlings under control conditions over a period of 24 h (Fig. 1d), the gene is inducible in response to diverse abiotic stresses such as; salinity (12 to 15 fold), high temperature (8 to 10 fold), drought (20 to 22 fold) and ABA (14 to 16 fold) (Fig. 1e - h).

### Generating transgenic *O. sativa* cv IR64 over-expressing *OsHBP1b*

To raise the transgenic rice, *OsHBP1b* was cloned into plant transformation vector pCAMBIA1304, using gene-specific forward and reverse primer pairs having specific restriction sites (Additional file 5: Table S1). For rice transformation, a highly efficient and reproducible *Agrobacterium tumefaciens*-mediated rice transformation protocol has been followed which has been standardized and published by our group, where mature seeds are taken as explants (Sahoo et al. 2011). Growing callus was transformed with positive *OsHBP1b*-recombinant *A. tumefaciens*. Transgenic plants were selected on MS media having 50 mg/L hygromycin. The transgenic plants which developed proper root in hygromycin-containing MS media were screened by tissue PCR with a combination of gene-specific forward and vector specific reverse primer pair (Additional file 5: Table S1 and Additional file 1: Figure S1a). For negative and positive controls, genomic DNA from wild type rice (WT; non-transgenic) and pCAMBIA1304-*OsHBP1B* plasmid respectively were taken as a template. Out of 89 plants regenerated, 17 (eight lines are shown here) were found to be positive as they showed the desired amplicon from the genomic DNA (Additional file 1: Figure S1a). Southern blot analysis confirmed the presence of a single copy of the transgene in two lines viz. L2 and L7 (Additional file 1: Figure S1b) which were then used for further experiments. Quantitative real-time PCR and western blot analysis revalidated the transgenic nature of these two lines with 4–5 fold higher accumulation of the *OsHBP1b* transcript and more expression of protein than the WT (Additional file 1: Figure S1c and Additional file 1: Figure S1d).

### Transgenic rice plants over-expressing *OsHBP1b* tolerate high level of salinity and maintain chlorophyll and ROS levels

**Fig. 2:**
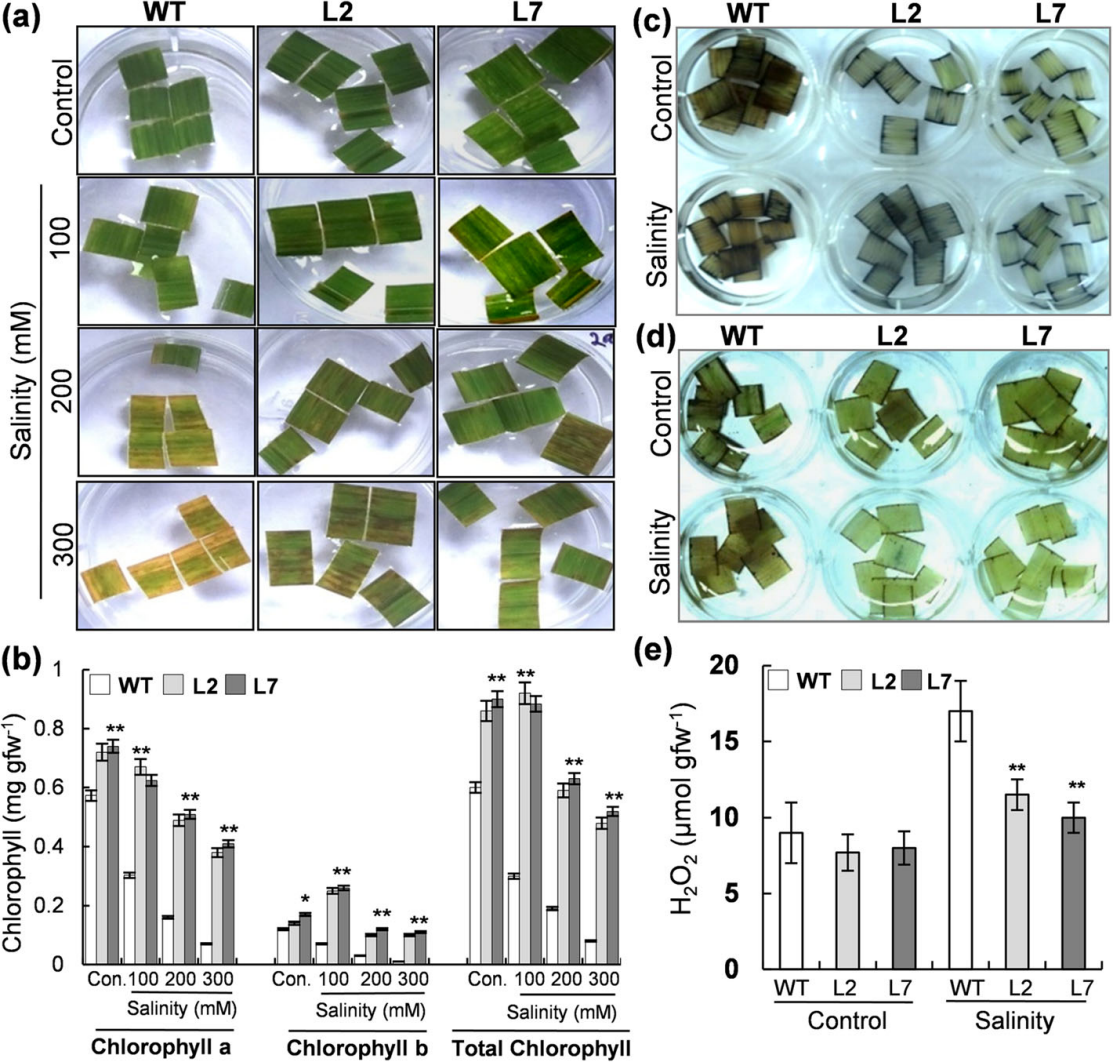
Leaf segment-assay showing NaCl-induced senescence in rice leaves through analysis of chlorophyll and ROS (O_2_^−^ and H_2_O_2_) content. **a** *OsHBP1b* over-expressing plant (L2 and L7) and WT plant leaf segments were kept in control (no NaCl) or different concentrations (100, 200 and 300 mM) of NaCl solution and pictures were taken after 72 h. The L2 and L7 leaves showing delayed leaf senescence as compared to the wild type leaves. **b** Chlorophyll contents (Chl-*a*, Chl-*b* and total Chl) in WT and transgenic rice leaf segments after 72 h of NaCl treatment. **c** Constitutive and salinity induced levels of O_2_^−^ were measured by NBT staining in WT and. **d** Salinity- induced H_2_O_2_ content were measured by DAB staining in WT and *OsHBP1b* over-expressing plants. **e** H_2_O_2_ content in WT and *OsHBP1b* over-expressing plants measured through spectrophotometer. Error bars show standard error from triplicate experiments﻿. Single asterisk show significant difference at *p* < 0.005 and double asterisk at *p* < 0.001

To assess the relative tolerance of *OsHBP1b* over-expressing rice plants towards salinity stress, quick testing was carried out using leaf segments of WT and the transgenic lines (for ease, these lines are henceforth referred as L2 and L7 in the manuscript). Leaf segments kept in NaCl solution confirmed early bleaching in the WT tissues than L2 and L7 plants (Fig. 2a). It was visibly seen that the L2 and L7 leaf segments could stay green by retaining a higher level of chlorophyll up to 72 h NaCl (300 mM) stress, unlike WT plants which showed very early bleaching (Fig. 2a). Under NaCl stress, the loss of chlorophyll in the WT plants was higher than L2 and L7 plants (Fig. 2b). It was observed that 300 mM NaCl treatment caused ~ 80% decrease in total chlorophyll level in the WT as compared to the control plants, while, barely 35–40% decrease was observed for the *OsHBP1b* over-expressing plants.

We examined the status of superoxide (O_2_^−^) and hydrogen peroxide (H_2_O_2_) in the WT, L2 and L7 transgenic lines through NBT and DAB staining, under control as well salinity-imposed condition (200 mM NaCl). The accretion of O_2_^−^ (Fig. 2c) and H_2_O_2_ (Fig. 2d) under control and salt stress was seen to be considerably less for the L2 and L7 than the WT plants. Spectrophotometric analysis for H_2_O_2_ measurement also revealed that the level of H_2_O_2_ in the L2 and L7 transgenic lines is lower (~ 33%) than the WT under NaCl stress. However, there was an insignificant difference in the level of H_2_O_2_ in WT and transgenic lines under control conditions (Fig. 2e).

### Transgenic rice plants over-expressing rice *HBP1b* can tolerate high salinity by maintaining physiological and enzymatic homeostasis

**Fig. 3:**
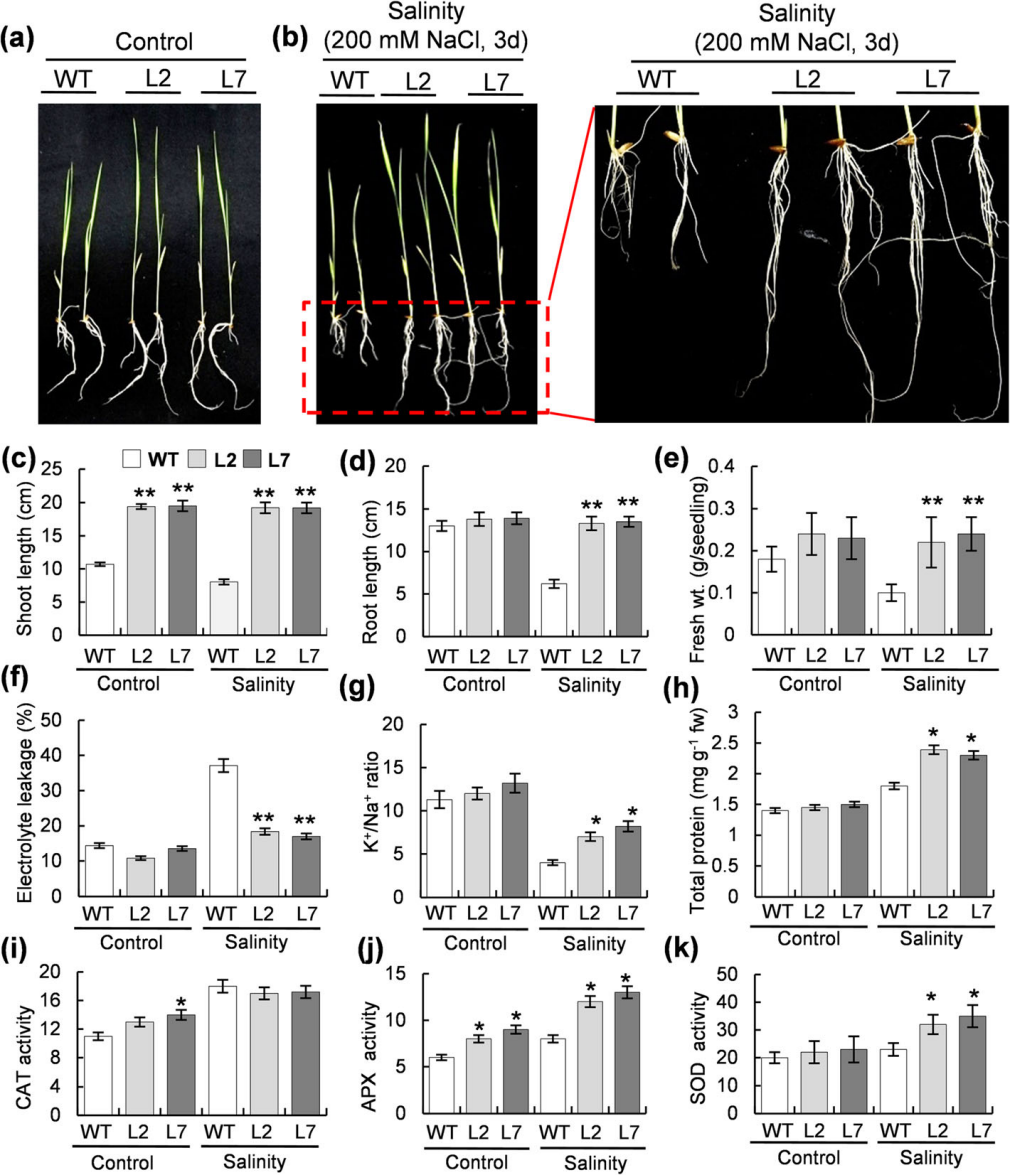
Evaluation of salt stress tolerance of *OsHBP1b* over-expressing rice seedlings through phenotypic, physiological and antioxidant enzyme analysis. **a** Photograph of WT and *OsHBP1b* over-expressing plants under control (non-stress) conditions. **b** Photograph of WT and *OsHBP1b* over-expressing plants after 72 h of salt stress to 7 d old seedlings. Enlarged view of root morphology has been shown on the right. Note that a prominent difference in root morphology was observed where *OsHBP1b* over-expressing plants have longer roots with more secondary branches as compared to the WT under salinity. Bar diagrams showing (**c**) shoot length, (**d**) root length, (**e**) fresh weight, (**f**) electrolyte leakage, (**g**) K^+^/Na^+^ ratio, (**h**) protein content, (**i**) CAT activity, (**j**) APX activity and (**k**) SOD activity measured in leaves of seedlings after 7 days of salinity stress. The data represent means ± SE of three biological triplicates. Single asterisk show significant difference at *p* < 0.005 and double asterisk at *p* < 0.001

Seven days old T_2_ generation rice seedlings were transferred to hydroponic solution containing 200 mM NaCl for 3d. Morphological indicators were analysed firstly to check the salinity response of *OsHBP1b* over-expressing lines. Although, there was a difference in shoot length of WT and *OsHBP1b* over-expressing seedlings, no significant changes were observed in their root length and root phenotype under control conditions (Fig. 3a). L2 and L7 transgenic lines showed a robust root system with more length and higher number of adventitious roots which grew thicker and longer than the wild type plants under salinity (Fig. 3b and d). Under salinity, shoot length and fresh weight of the L2 and L7 lines were also found to be increased than the WT plant (Fig. 3c and e). Electrolyte leakage (EL), a primary indicator of membrane damage under stress (Verslues et al. 2006), was measured in the *OsHBP1b* over-expressing transgenic plants. Under control conditions, we could not get significant differences in the electrolyte leakage between WT and the transgenic lines (Fig. 3f). On the other hand, under salinity stress, the over-expressing lines showed a comparatively lower (50%) EL than the WT plants (Fig. 3f). To check if *OsHBP1b*-mediated increase in salt stress tolerance resulted by the mitigation of the lethal Na^+^ ions, the Na^+^ and K^+^ contents in the WT, L2 and L7 plants was measured. The K^+^/Na^+^ ratio was found to be higher in the leaves of the transgenic plants than the wild type (Fig. 3g), whereas, no significant change in K^+^/Na^+^ ratio was observed in WT and over-expressing lines under control conditions (Fig. 3g).

We measured the total protein level and carried out antioxidant enzyme activity assays in control as well as salinity-treated leaves from WT and *OsHBP1b* over-expressing seedlings. Under control conditions, WT, L2 and L7 plants showed a comparable level of protein, whereas, under salinity, L2 and L7 lines showed ~ 20% higher protein content than the WT plants (Fig. 3h). Though CAT activity was found to remain largely unchanged in transgenic lines and WT seedlings under salinity stress (Fig. 3i), increased APX (~ 43%) and increased SOD (20–30%) activity was quite evident under similar conditions (Fig. 3j and k). These results also indicate that the expression levels of *OsHBP1b* in rice are correlated with salinity tolerance in transgenic lines as mediated via maintenance of cellular physiology and enzymatic homeostasis.

### Stomatal functions and organelle structure contribute towards salinity tolerance in the *OsHBP1b* over-expressing plants

**Fig. 4:**
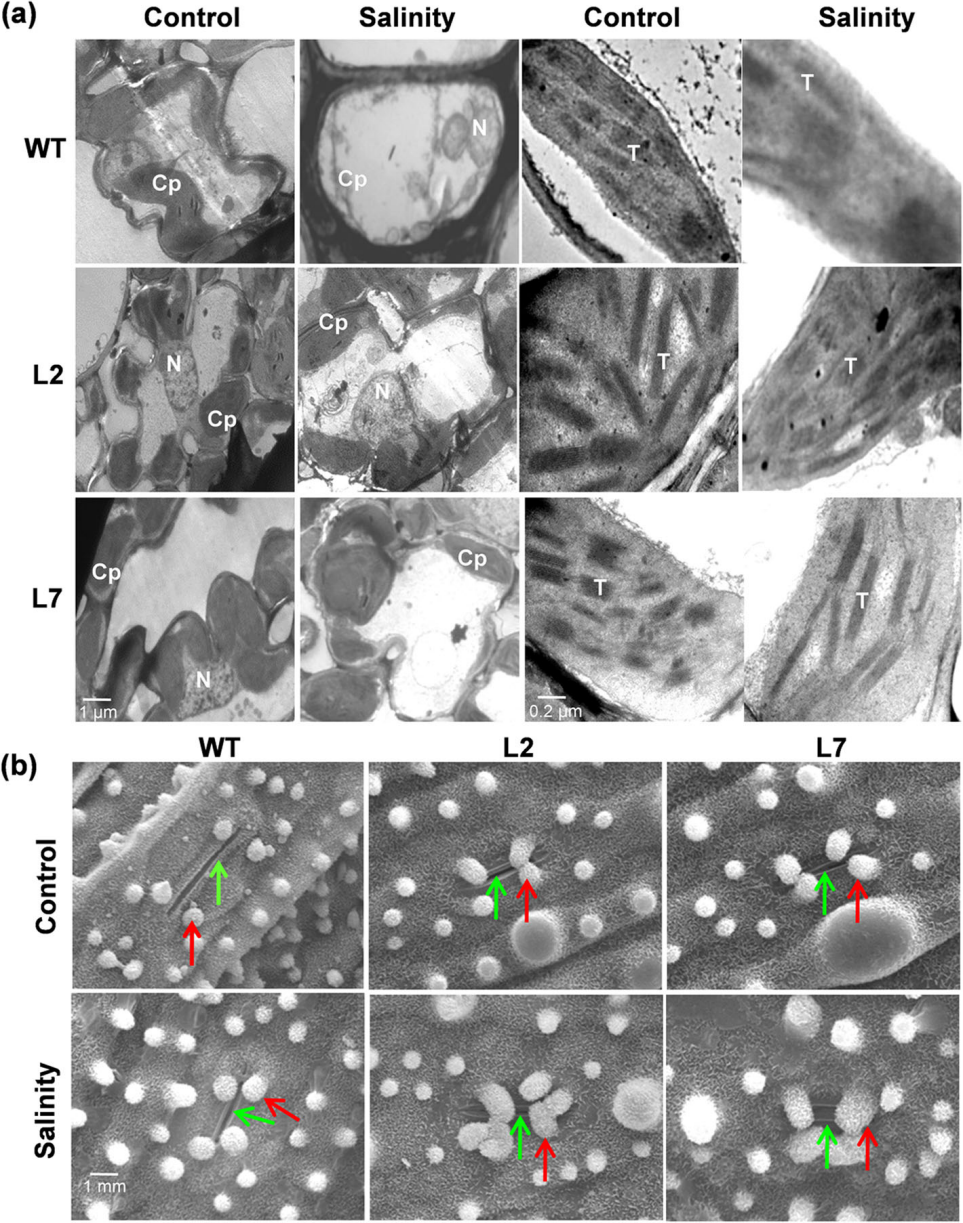
TEM and SEM-based anatomical analysis of WT and *OsHBP1b* over-expressing plants under control and salinity stress. **a** Structure of complete cell of WT (1st row) and *OsHBP1b* over-expressing plants (2nd row) under control (1st column) and after 200 mM NaCl treatment (2nd column) as observed under TEM. Third and fourth column are showing the structure of a single chloroplast and organisation of thylakoid under control and salinity conditions in WT and over-expressing plants. **b** SEM picture showing the structure of stomata in WT and *OsHBP1b* over-expressing plants under control and salinity stress. Note that under salinity, the stomata of *OsHBP1b* over-expressing plants are protected. Cp, chloroplast; N, nucleus; T, thylakoid. Green and red arrows indicate stomata and silica cell respectively

The *OsHBP1b* over-expressing lines showed healthy cellular structure with intact chloroplasts and proper thylakoid stacking under salt stress, unlike the wild type plants. In wild type plants, severe cell damage and distorted chloroplast and thylakoid stacking was found when leaf sections from salinity treated plants were analyzed through TEM (Fig. 4a). In WT and transgenic lines, a similar type of stomatal phenotype was observed under control conditions, whereas under salinity stress, the stomata of L2 and L7 transgenic lines were found to be largely closed and protected (Fig. 4b). These findings suggest enhanced tolerance of the transgenic plants towards salinity stress as compared to the WT plants via maintenance of healthy cellular organelles and protected stomata.

### Rice plants over-expressing *OsHBP1b* exhibit efficient photosynthesis and set viable seeds under salinity

**Fig. 5:**
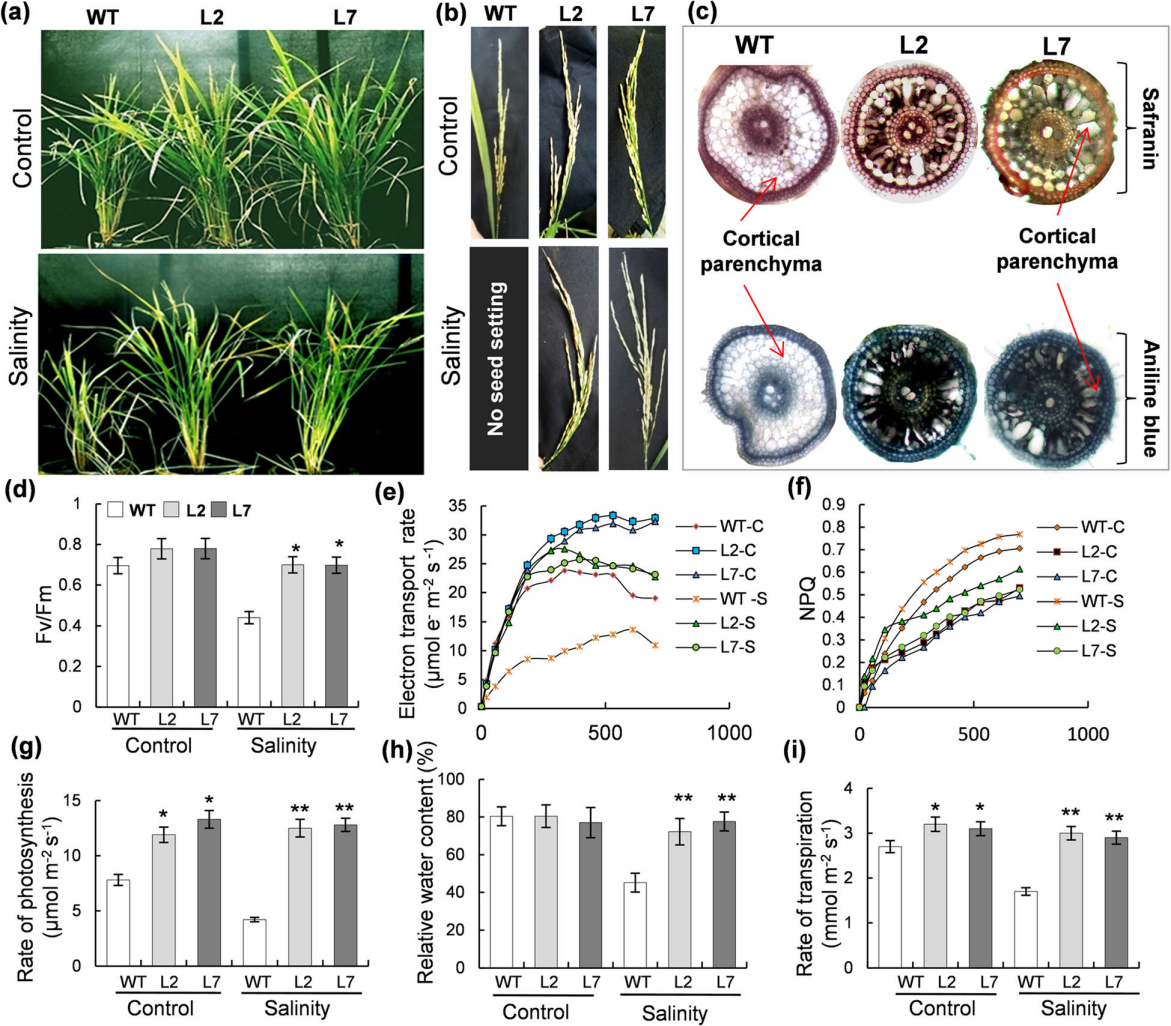
Assessment of physiological and morphological parameters of mature *OsHBP1b* lines as compared to the WT under salinity. **a** Pictorial view of mature WT and *OsHBP1b* over-expressing plants. Note the better growth in terms of plant height, and number of leaves in the *OsHBP1b* over-expressing plants as compared to WT under both control and salinity conditions. **b** Panicle morphology of WT and *OsHBP1b* over-expressing plants. Note that under salinity, there was no panicle produced in the WT plants. **c** Anatomy of the root of WT and *OsHBP1b* over-expressing plants stained with safranin (upper panel) or aniline blue (lower panel) under control conditions. **d** Fv/Fm, (**e**) electron transport rate, (**f**) NPQ, (**g**) rate of photosynthesis, (**h**) relative water content and (**i**) rate of transpiration, in *OsHBP1b* over-expressing plants as compared with the WT. Error bars show standard error from triplicate experiments. ﻿Single asterisk show significant difference at *p* < 0.005 and double asterisk at *p* < 0.001

Phenotypic analysis revealed a better morphology of *OsHBP1b* over-expressing plants under control as well as 200 mM of salinity stress (Fig. 5a). The plant height for the transgenic lines was found to be higher than the WT under control as well as salinity conditions (Fig. 5a). Under non-stress conditions, both WT and transgenic plants grew well and set seeds upon maturity. However, under salinity, the WT plants could not grow well and failed to set flower even after recovery (under conditions of adequate water availability). On the other hand, the transgenic plants could flower normally and set seed following salinity treatment and subsequent recovery (Fig. 5a and b). Analysis of the root anatomy of WT and transgenic lines grown under control condition showed that the transgenic lines possess an enlarged stele and bigger cortical parenchyma cells with an expanded cortex (Fig. 5c, upper panel). Also, aniline blue staining revealed that the roots of transgenic lines accumulate a good amount of callose in the cortex, unlike the WT roots (Fig. 5c, lower panel).

Further, various photosynthesis related parameters for the WT and transgenic lines under control and salinity stress were studied using IRGA. Under control conditions, no significant variations were observed between WT and the transgenic lines in terms of their Fv/Fm ratio, Electron transport rate (ETR), Non-photochemical quenching (NPQ) and CO_2_ assimilation (Fig. 5d-g). A drastic decrease in Fv/Fm (~ 32%), ETR (50 to 65%) and CO_2_ (~ 70%) assimilation were observed in WT as compared to the transgenic lines under salinity conditions (Fig. 5d, e, and g). However, salinity did not had any significant adverse effect on the transgenic lines, in terms of their Fv/Fm ratio, ETR and CO_2_ assimilation. As expected, the non-photochemical quenching (NPQ) in the WT plants increased under salinity as compared to the transgenic lines (Fig. 5f). Furthermore, the relative water content and the rate of transpiration was found to be higher (43 and 49% respectively) in the transgenic lines than the WT plants under salinity conditions, while there was no change observed between the WT and transgenic lines under normal growth conditions (Fig. 5h and i).

### The transgenic *OsHBP1b* over-expressing lines exhibit altered expression of photosynthesis- and stress-related genes

**Fig. 6:**
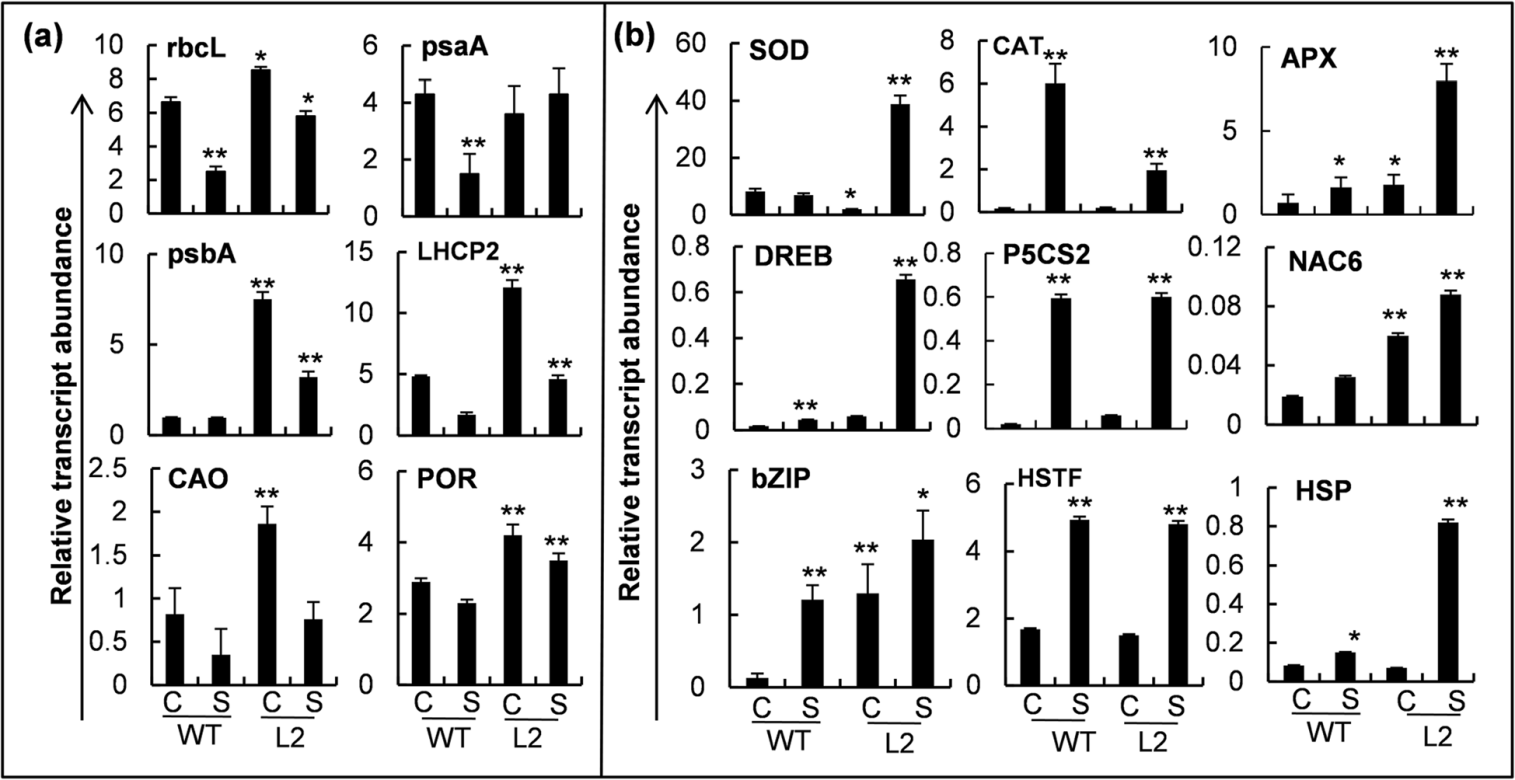
Transcript abundance analysis for the various photosynthesis and stress-related genes in WT and *OsHBP1b* over-expressing plants under control and salinity. **a** Relative expression analysis of selected photosynthesis related genes such as ribulose bisphosphate carboxylase/oxygenase (RbcL**)**, Photosystem I P700 chlorophyll a apoprotein A1 (PsaA), Photosystem II P680 chlorophyll a apoprotein A1 (PsbA), Light harvesting complex protein 2 (LHCP2), chlorophyllide *a* oxygenase (CAO) and P450 oxidoreductase (POR) in WT and *OsHBP1b* over-expressing plants. **b** Relative expression analysis of selected stress responsive gens (SOD, superoxide dismutase; APX, ascorbate peroxidise; CAT, catalase; bZIP, leucine zipper transcription factor; HSTF, heat shock transcription factor; HSP, heat shock protein; MT, metallothionine; DREB, dehydration responsive element binding protein; NAC TF, NAC domain containing transcription factors; P5CS, 2-pyrroline-5-carboxylate synthetase-2) in WT and *OsHBP1b* over-expressing plants. Expression analysis was done by quantitative real time PCR method. Expression of eEF gene was taken as reference and transcript abundance was calculated using 2^-ΔCT^ method. Error bars show standard error from triplicate experiments. ﻿Single asterisk show significant difference at *p* < 0.005 and double asterisk at *p* < 0.001

To analyse the expression of photosynthesis- and stress-related genes, we performed quantitative real-time PCR for a few selected genes in rice seedlings under control and salinity conditions. Under control conditions, significantly higher levels of expression was observed for most of the selected photosynthesis-related genes such as; rbcL (~ 1.3fold), psbA (~ 8 fold), LHCP2 (2.5 fold), CAO (~ 1.7 fold) and POR (~ 3.3 fold) in the *OsHBP1b* over-expressing plants as compared with the WT (Fig. 6a). Only psaA gene showed an insignificant difference in the expression pattern when compared to WT. Most of the selected genes in both types of plants showed decreased expression under salinity, but it was evident that even after salt treatment, the level of expression of all the selected photosynthesis-related genes in *OsHBP1b* over-expressing lines was comparable to the level of expression of the selected genes in the salt-untreated (control) WT plant (Fig. 6a).

Apart from photosynthesis-related genes, we also investigated the expression kinetics of various stress-related genes in the *OsHBP1b* over-expressing lines. Under control conditions, there were three types of expression patterns observed viz. (i) most of the genes (ascorbate peroxidase; APX, NAC6 TF, DREB TF, Proline biosynthetic gene; P5CS2 and bZIP TF) showed increased expression in *OsHBP1b* over-expressing lines, (ii) one of the genes (SOD) showed decreased expression in *OsHBP1b* over-expressing lines and (iii) for some of the other genes (catalase; CAT, heat shock TF; HSTF and heat shock protein; HSP), no change in expression was observed between WT and *OsHBP1b* over-expressing lines (Fig. 6b). However, after salinity treatment, there were higher levels of expression (SOD, ~ 4 fold; APX, ~ 5 fold; DREB, ~ 7 fold; NAC, ~ 2.5 fold, bZIP, ~ 1.5 fold and HSP, ~ 4 fold) observed in *OsHBP1b* over-expressing lines, except CAT, P5CS2 and HSTF (Fig. 6b). A decreased (3.5 fold) level of CAT transcript was observed in the *OsHBP1b* over-expressing lines as compared with WT under salinity stress (Fig. 6b). No change in P5CS2 and HSTF transcripts were evident in *OsHBP1b* over-expressing lines as compared to the WT, under salinity (Fig. 6b).

### Over-expression of *OsHBP1b* in transgenic rice plants enable them to tolerate multiple abiotic stresses

Apart from salinity, we have analysed the performance of *OsHBP1b* over-expressing lines under diverse abiotic stresses by exposing the plants to drought, high temperature, and ABA. It was evident that the following recovery after drought (PEG) and high-temperature treatment, the transgenic lines over-expressing *OsHBP1b* performed better having higher shoot length, root length and fresh weight (Additional file 2: Figure S2 a-c and Additional file 3: Figure S3 a-c). In both the stress treatments, the root morphology was found to be more favourable in the transgenic lines as compared to the WT where more number of secondary and adventitious roots were evident (Additional file 2: Figure S2a and Additional file 3: Figure S3a). In order to assess their photosynthesis capacity, we checked the Fv/Fm in these plants and found a higher Fv/Fm value in the *OsHBP1b* over-expressing lines as compared to the WT, under both drought and high temperature stress (Additional file 2: Figure S2d and Additional file 3: Figure S3d). Significant increase in APX (~ 37% in drought and 35% in high temperature) and SOD (20% in drought and 18% in high temperature) activity was evident in the *OsHBP1b* over-expressing lines as compared to the WT, under drought and high temperature conditions (Additional file 2: Figure S2f, S2g, Additional file 3: Figure S3f and Additional file 3: Figure S3g). Under drought stress condition, the activity of catalase did not change significantly in WT and *OsHBP1b* over-expressing lines, whereas, high temperature could induce the CAT activity up to 31% (Additional file 2: Figure S2e and Additional file 3: Figure S3e). Since it is well established that bZIP proteins are strongly induced by ABA (Liu et al. 2014), the responsiveness of the *OsHBP1b* over-expressing and WT lines was also studied after treatment with 5 and 10 μM of ABA. It was observed that, under 5 μm of ABA, the WT plants possessed a lesser shoot and root length than the *OsHBP1b* over-expressing lines, whereas, *OsHBP1b* over-expressing lines could grow normally without any inhibition of shoot and root growth under similar conditions (Additional file 4: Figure S4a and S4b). The fresh weight of the WT plant was also found to be less in comparison to the *OsHBP1b* over-expressing lines under 5 μM of ABA treatment (Additional file 4: Figure S4c). It was also evident that 10 μM of ABA affected the shoot and root growth severely, unlike the *OsHBP1b* over-expressing lines which germinate and grow better even upon 10 μM of ABA treatment (Additional file 4: Figure S4d and S4e). The fresh weight of the WT seedlings was also drastically decreased upon ABA treatment, as compared to the *OsHBP1b* over-expressing lines (Additional file 4: Figure S4f). These results clearly suggest that the expression level of *OsHBP1b* in rice is certainly linked with tolerance to multiple abiotic stresses which makes the plant tolerant to various stresses, unlike the WT plants.

## Discussion

HBP1b protein is classified under bZIP transcription factors family due to its DNA binding specificity. OsHBP1b contains a highly conserved bZIP domain as evidence of a bZIP family protein (Busch and Sassone-Corsi 1990; Lakra et al. 2015). This protein has the highest affinity for ‘ACGTCA’ hexamer motif in the promoter regions of histone genes, and are reported to be participated in the regulation of histone gene transcription (Mikami et al. 1989; Tabata et al. 1991). We have cloned the full length *OsHBP1b* gene in the plant transformation vector pCAMBIA 1304. Sequence analysis of OsHBP1b protein revealed that it possesses more than 94% similarity with the reported HBPs of barley and wheat (Lakra et al. 2015). OsHBP1b protein has been nuclear localized (Lakra et al. 2015) which was also in agreement with other reports (Mikami et al. 1989).

*OsHBP1b* is a *Saltol* QTL-localized gene found on chromosome number 1 in the rice genome. Previous reports have documented the constitutive and salinity-induced expression patterns for the various genes localized within the *Saltol* QTL (Soda et al. 2013; Nutan et al. 2017), which revealed *OsHBP1b* gene to be induced under salinity in rice seedlings. This observation led us to hypothesize that *OsHBP1b* might have a key function in rice salt stress tolerance. Subsequently, this hypothesis got strong support from the observation when the seedlings from the salt-tolerant genotype (Pokkali) showed elevated constitutive expression of *OsHBP1b* transcripts than the sensitive IR64 with further differential accumulation of transcripts under salinity stress (Lakra et al. 2015). Further analysis of the upstream and genic region of OsHBP1b confirmed significant differences in the single nucleotide polymorphisms (SNPs) and insertion-deletions (InDels) patterns in the contrasting genotypes IR64 and Pokkali (Additional file 6: Table S2). It is a fact that the bZIP TFs are indispensable in plant growth and development and their expression can be induced in response to abiotic and biotic stresses (E et al. 2014). In the present study, we observed that *OsHBP1b* is expressed during all the developmental stages of rice (Fig. 1a). However, this gene showed differential expression in various plant tissues when analysed using the publicly available microarray database and subsequent validation by quantitative real-time PCR (Fig. 1b and c). Several reports have shown that the expression of bZIP transcription factor genes gets altered in response to various abiotic stresses (Gao et al. 2011; Liu et al. 2014). Our results are in accordance with these reports where we found the induced expression of *OsHBP1b* transcript in response to various abiotic stresses such as salinity, high temperature, drought or ABA (Fig. 1e-h). These results motivated us to investigate the function of rice *HBP1b* towards enhancing abiotic stress tolerance in rice by over-expressing the gene using a strong promoter (CaMV35S).

In the present study, we over-expressed *OsHBP1b* in a high yielding salt-sensitive rice IR64, with the purpose to dissect out the role of this gene towards multiple abiotic stress tolerance. It was found that *OsHBP1b* over-expressing single copy lines possess 4–5 fold higher expression of *OsHBP1b* transcript (Additional file 1: Figure S1) confirming the transgene functionality. It is well known that even though the response of plants towards abiotic stress is a multigenic trait, salt tolerant transgenic plants could be developed by introducing a single gene (Singla-Pareek et al. 2001; Tripathi et al. 2016; Soda et al. 2018). Published literature has also shown that the plants ectopically-expressing bZIP transcription factors exhibit a positive effect on their growth, development and salinity tolerance (Gao et al. 2011; Liu et al. 2014; E et al. 2014; Lakra et al. 2015). Our findings are in agreement with these reports. We found better germination capacity, higher shoot growth, and higher fresh weight in *OsHBP1b* over-expressing lines than the WT, under control as well as salinity conditions (Fig. 3a, b, c, d and e). Larger parenchyma cells, longer roots, higher numbers of root branches were reported being responsible for providing dehydration stress tolerance by having more water holding capacity in rice plants (Ambavaram et al. 2014). In agreement to this study, we found better root morphology with longer root and more secondary branches in the transgenic plants under various abiotic stress conditions, in comparison with the WT plants (Fig. 3a, b and d, Additional file 2: Figure S2a and S2b, S3a and S3b and Additional file 4: Figure S4a and S4b). In addition, under control conditions, we also found the larger cortical region with larger parenchyma cells with accumulated callose (Fig. 5c) which might be providing limited access to ions and hence, providing salinity tolerance in the transgenic plants. Our finding of larger cortical parenchyma in transgenic plant is corroborated with a recent finding in wheat showing the genotype having larger root cortical cell required less energy to penetrate the hard soil and thus less translocation of carbohydrate to root is required which remains critical for plant survival under stress (Colombi et al. 2019). Larger metaxylem, which we have observed in transgenic rice root is reported to be a positive characteristic helping the plant under drought stress (Kadam et al. 2015). Various reports have stated the suberization, lignification and callose deposition in root cell under salinity stress to prevent the entry of harmful ions in the cell (Koyro 1997; Cui and Lee 2016; Hunter et al. 2019). Even though introduction of bZIP TF led to enhanced stress tolerance in plants, the mechanism of tolerance primarly remains unknown. Furthermore, characterization and role (if any) of OsHBP1b in stress response in rice has not been explored yet. This encouraged us to perform further biochemical and physiological analysis of the *OsHBP1b* over-expressing rice, in response to salt stress as well as under other major abiotic stress conditions.

Treatment of WT and transgenic leaf segments in 100, 200 and 300 mM NaCl, revealed early senescence of the WT leaf as compared to the *OsHBP1b* over-expressing plants (Fig. 2a). Further, the reduction in chlorophyll contents in the *OsHBP1b* transgenic lines was lesser (15–25%) than the wild type counterparts (Fig. 2b). These results show a positive relationship between the expression of *OsHBP1b* and salt stress tolerance in leaf tissues. It has been established that the accumulation of ROS is connected to physiological balance and any fluctuations in ROS level can disturb the normal function of cellular machinery (Miller et al. 2010; Li et al. 2013). So, we checked the ROS level in plants by histochemical staining with NBT and DAB, which verified that under salt stress, the *OsHBP1b* over-expressing plants accrue less reactive oxygen molecules (O_2_^−^ and H_2_O_2_) than wild type plants (Fig. 2c and d). It is also known that the level of ROS plays a crucial role in stress response in plant (Székely et al. 2008). For detoxification of ROS produced during stress, plants build up a multifaceted antioxidant arrangement, where several antioxidant proteins have a significant contribution towards protecting plant/cells against oxidative burst (Jaleel et al. 2009; Miller et al. 2010; Kumar et al. 2013). Under salinity, the activity of APX and SOD was found to be higher in the OsHBP1b over-expressing plants than the wild types (Fig. 3j and k). Our work indicated that enhanced ROS scavenging through antioxidant enzymes might be an essential part of salinity protection machinery in the developed OsHBP1b transgenic lines. In agreement to our previous study (Lakra et al. 2015); insignificant change in CAT activity (Fig. 3i) under salt stress condition indicated the least participation of CAT in salinity tolerance system. Moreover, reports confirmed the induction of ROS scavenging genes after ectopic expression (Wu et al. 2008), representing the connection of transgene in the transcriptional regulation of antioxidant enzyme pool. In agreement with this, we have also observed induced expression of CAT, APX and SOD transcripts under salinity conditions (Fig. 6). Reports have claimed that under stress conditions, total protein level and percentage of electrolyte leakage changes drastically (Song et al. 2011; Gulen and Eris 2003). We observed a reduction in the percentage of electrolyte leakage in the *OsHBP1b* over-expressing transgenic plants (Fig. 3f), which is known to participate in plant stress tolerance mechanism in a constructive manner (Song et al. 2011). We observed a higher K^+^/Na^+^ and higher protein content in the *OsHBP1b* over-expressing lines when compared with the wild type plants (Fig. 3g and h), which are also positive indicators of stress tolerance. Our result are in corroboration with other findings where enhanced tolerance in transgenic rice overexpressing a bZIP transcription factor (OsbZIP71) has been reported to confer salinity and drought tolerance in rice (Liu et al. 2014). Role of bZIP TF in stress tolerance has also been established by RNAi technology where silencing of similar bZIP1 domain containing gene PtabZIP1, AtbZIP1 and SibZIP1 from poplar, Arabidopsis and tomato results in enhanced sensitivity towards salinity and drought which further affects the overall plant growth and development (Sun et al. 2012; Dash et al. 2017; Zhu et al. 2018).

It has already been proven that salinity stress has a negative effect on the structure of the plant organelles (Pareek et al. 1997; Xu et al. 2008; Salama et al. 2012). Here, we found that over-expression of *OsHBP1b* could protect the cellular structure by maintaining the organellar membrane and thylakoid stacking, unlike the WT plants, where all the organelles including cell wall showed severe damage upon 200 mM NaCl treatment (Fig. 4a). Stomata of the *OsHBP1b* over-expressing lines were found to be protected which may contribute towards controlling the rate of transpiration under salinity conditions (Fig. 4b). These result are in harmony with the previous reports by Gao et al. (2011), where the closing of stomata was evident upon stress treatment.

Several studies have demonstrated that plants under abiotic stress conditions downregulate the expression of genes involved in photosynthesis and carbohydrate metabolism (Seki et al. 2002; Wong et al. 2006). Furthermore, transcription factors are also found to be involved in photosynthetic improvement when over-expressed (Saibo et al. 2009). In agreement with Saibo et al. (2009), we found increased chlorophyll fluorescence parameters, decreased non-photochemical quenching and increased photosynthesis activity in the *OsHBP1b* over-expressing lines as compared with the WT, under control as well as salinity conditions (Fig. 5). Furthermore, a recently published report showed that coordinated regulation of photosynthesis in rice could increase the environmental stress tolerance and yield related parameters (Ambavaram et al. 2014). Importantly, higher chlorophyll content and plant biomass were evident in the *OsHBP1b* over-expressing rice under salinity conditions (Fig. 2b), which are the major achievements of this study.

It is a well-established fact that TFs are present in all the organisms as they are the integral part of gene expression and regulation (Ashraf and Harris 2013; Joshi et al. 2016). Recently, the role of numerous TFs involved in regulation of the photosynthesis related-genes has been described (Saibo et al. 2009). Furthermore, it has been reported that bZIP transcription factor such as; LOG HYPOCOTYL 5 (HY5) is mainly involved in the regulation of Chl a/b binding gene expression, though it may also have an important contribution in abiotic stress tolerance (Saibo et al. 2009). Additionally, it has also been shown that this bZIP TF controls Rubisco small subunit (rbcS1A) gene expression (Chattopadhyay et al. 1998; Lee et al. 2007). In agreement with these results, we also observed an increased expression of various photosynthesis related genes (rbcL, psbA, LHCP2, CAO and POR) in the *OsHBP1b* over-expressing plants in comparison with WT (Fig. 6a). Furthermore, it has also been revealed that the expression of these photosynthetic genes decreased under salinity stress in WT plants where as *OsHBP1b* over-expressing lines maintained the gene expression to the level of WT control plants, even under salinity (Fig. 6a).

Apart from maintained photosynthesis rate, abiotic stress tolerance is an essential character to get higher plant yield under unfavourable environmental conditions. Modulation in the expression of stress-related genes is another way to develop stress tolerance in the plants. As it is known that abiotic stress tolerance is a multigenic trait, non-regulatory genes related to stress tolerance do not always meet the aim to get abiotic stress tolerant plants after over-expression. To overcome this, TFs that regulate several genes have often used to increase stress tolerance in a broader response (Nelson et al. 2009). Many transcription factors such as; bZIP (Zhang et al. 2008), NAC (Hu et al. 2006), MYB (Vannini et al. 2004) and zinc finger (Kim et al. 2001), have been found to be involved in providing multiple stress tolerance by regulating various important functions and manipulating many stress-responsive genes. Moreover, some researchers have established that bZIP TFs help to regulate stress-related genes under control as well as abiotic stress situations (Huang et al. 2010; Yáñez et al. 2009; ZG et al. 2014). Interestingly, we also found altered expression of various stress-related transcripts including antioxidant genes; heat stress related genes and drought stress related genes, in *OsHBP1b* over-expressing lines under stress conditions (Fig. 6). These results suggested that over-expression of *OsHBP1b* was able to alter the expression of the stress-responsive genes and are positively involved in providing higher photosynthesis and stress tolerance under salinity.

As abiotic stresses are interrelated to each other, we further decided to verify the drought, high temperature and ABA responsiveness of the *OsHBP1b* over-expressing lines with a comparison to its WT counterpart. Our results clearly suggested that the *OsHBP1b* over-expressing lines have a better phenotype, antioxidant activity and photosynthesis rate under drought and high-temperature stress conditions (Additional file 2: Figure S2 and Additional file 3: Figure S3). Moreover, bZIP transcription factors have been recognized as important players in ABA signalling pathway of abiotic stress (Liu et al. 2014). In accordance, we have found better shoot and root growth of *OsHBP1b* over-expressing lines even with 10 μM of ABA treatment, while at this particular concentration of ABA, WT plants could not even germinate (Additional file 4: Figure S4). This proves a crosstalk between the stress response and harmones in plants (Karan et al. 2009; Nongpiur et al. 2012).

## Conclusions

The function of bZIP subfamily in rice is less explored. Here, we have investigated the possible role of bZIP subfamily protein (OsHBP1b) in rice and, taken together, our results describe the importance of *OsHBP1b* in relation to stress tolerance and plant yield. Over-expression of *OsHBP1b* in rice, under the control of CaMV35S promoter, signifies its own role in stress management by minimizing the level of ROS, increasing antioxidant enzyme activity, maintaining organelle structure, increasing photosynthesis and modulating the level of photosynthesis and stress-related transcripts. Furthermore, ABA receptiveness of the generated *OsHBP1b* transgenic rice plants suggests that the stress tolerance behaviour in these lines is related to the ABA signal transduction pathway. Nevertheless, the reality that *OsHBP1b* over-expression noticeably showed minimum impact on grain filling and panicle length of rice under salinity suggests that this gene could be used in other plants species to increase their productivity under salinity and other unfavourable environmental situations.

## Methods

### Plant material and growth conditions

Rice *(Oryza sativa* L., cv. IR64) was used for the expression analysis of *OsHBP1b* under control and various abiotic stress conditions. Rice seeds were surface sterilized and grown by following the protocol as mentioned in Lakra et al. (2015). 7 days old plants were exposed to salinity, drought, high temperature and ABA stress and use for further analysis using standard protocols, mentioned in following section. For further study of various growth parameters and seed harvesting, 25 days old T_1_ or T_2_ *OsHBP1b* over-expressing rice lines were transferred to green house.

### Stress treatments

In order to determine the transcript abundance, 7 d old rice seedlings grown hydroponically under control conditions were transferred to a solution of 200 mM NaCl up to 24 h. Samples were collected from 0 h, 0.5 h, 12 h, and 24 h after stress treatment. For high temperature stress, rice seedlings were transferred to a growth chamber maintained at  45 ºC and samples were harvested after 0 h, 0.5 h, 3 h and 6 h. Similarly, for drought stress, the seedlings grown on 5 % PEG were used and for ABA seedlings were treated with 10 μM ABA and samples were harvested after 0 h, 0.5 h, 12 h and 24 h of stress imposition. A set of seedlings were alwayes kept under control conditions for the same duration. For various morphological and physiological parameters (shoot length, root length, fresh weight, electrolyte leakage, K^+^/Na^+^ ratio, total protein and antioxidant enzymes activity) of *OsHBP1b* over-expressing rice in response to salinity, 7 d old T_2_ rice seedlings grown hydroponically under control conditions were transferred to solution of 200 mM NaCl up to 3 d. Samples were collected after 72 h of stress treatment. Tissue-specific *OsHBP1b* expression and expression of *OsHBP1b* after transgene insertion were done by using 24 h NaCl treated plants samples. The expression of stress-related and photosynthesis-related genes was studied using 72 h stress-treated samples. For assessment of other parameters like organellar organization, stomatal structure, H_2_O_2_, superoxide level, and photosynthetic parameters and yield related parameters, 90 d old plants were treated with 200 mM NaCl solution for 15 days, followed by recovery with water. For the leaf segment assay, leaf segments (1 cm length) were incubated (for 72 h) with liquid Yoshida medium supplemented with 100 mM or 200 mM or 300 mM NaCl.

For the assessment of growth and physiological parameters of *OsHBP1b* over-expressing rice plants under multiple abiotic stresses, T_2_ rice seedlings were grown under control conditions inside a growth chamber for 7 d and subsequently, treated with either ABA (5 or 10 μM for 72 h) or PEG (5%, for drought stress for 72 h) or high temperature (45 °C for 12 h, subsequent recovery for 3 d) in a hydroponic setup.

### Transcript abundance analysis

The transcript abundance for *OsHBP1b* at different stages of development was analyzed using the publicly available database (https://www.genevestigator.com/gv/) using default parameters. The expression analysis of various genes was carried out by quantitative real-time PCR method as described earlier (Lakra et al. 2015; Nutan et al. 2017). Primer pairs used for transcript expression were given in Additional file 5: Table S1.

### Raising of transgenic rice plants over-expressing *OsHBP1b* and confirmation of transgenic lines

Cloning and transformation of *OsHBP1b* gene was done by following the earlier protocols where the expression of the gene is controlled by CaMV 35S constitutive promoter (Lakra et al. 2015). Callus induction, callus transformation, and plant regeneration from transformed callus were done by following the protocol of Sahoo et al. (2011). *OsHBP1b* over-expressing rice plants were selected on MS media having 50 mg/l hygromycin. The *OsHBP1b* over-expressing rice plants which grew properly in hygromycin-supplemented media were screened through tissue PCR with a combination of gene-specific forward and vector specific reverse primer pairs. Genomic DNA from wild type IR64 and pCAMBIA1304*OsHBP1b* plasmid were taken as negative and positive control respectively. 8 plants positive for the presence of the transgene are shown here as they amplified the desired band from the genomic DNA of the putative OsHBP1b over-expressing lines. Segregation analysis of T1 transgenic seeds were done in presence of hygromycin showing their single copy nature (Additional file 7: Table S3)**.** Transgenic nature of OsHBP1b over-expressing plants was also confirmed by Southern hybridization method as described by Sambrook and Russell (2001), where *Eco*RI was used to digest the genomic DNA and OsHBP1b was used as probe**.**

### Western blot analysis

The relative concentration of HBP1b proteins in the leaf extracts of WT and transgenic plants was analyzed by western blot analysis. The crude protein extracted in 50 mM K_2_PO4 buffer (pH 7.0) containing 1 mM PMSF was separated on a 10% SDS-PAGE and was transferred onto the activated immunoblot PVDF membranes (Sigma) with semi-dry blotting apparatus according to the provided instructions. To check the abundance of HBP1b protein, the blots were treated with protein-specific polyclonal antibodies (Lakra et al. 2015) separately and developed with a SuperECL Plus kit (Thermo Scientific, Pierce, USA), and the signal was exposed with X-ray film.

### Physiological and biochemical analysis for the transgenic plants

#### Na^+^ and K^+^ measurement

To determine the leaf Na^+^ and K^+^ content, 100 mg of tissues from NaCl-treated and untreated plants were digested with HNO_3_ (0.1%). Determination of K^+^ and Na^+^ content was done by atomic absorption spectrometer (Central Instrument Facility, Jawaharlal Nehru University) following the protocol of Lakra et al. (2015).

#### Electrolyte leakage

Analysis of electrolyte leakage was carried out following the protocol described in Lakra et al. (2015). 100 mg leaf samples were taken for this analysis and the formula used for measurement of relative electrical conductivity is: electrolyte leakage (%) = E1/E2*100 where E1 is electrical conductivity and E2 is total conductivity.

#### Chlorophyll estimation

Chlorophyll content in control and NaCl treated tissues was determined following the method of Arnon (1949) using UV/Vis spectrophotometer.

#### Determination of H_2_O_2_ and ROS levels

Level of reactive oxygen species (ROS) in the leaves was estimated through histochemical staining assay using NBT (nitro blue tetrazolium) as described by Lakra et al. (2015). In situ detection of hydrogen peroxide (H_2_O_2_) was performed by staining the tissue with DAB (Sigma-Aldrich) as suggested (Bindschedler et al. 2006). Steady-state levels of H_2_O_2_ in leaves were determined following FOX-1 method (Sahu et al. 2010).

#### Total protein measurement and activity assay of antioxidant enzyme

Total proteins extraction and antioxidant enzyme activity assays were performed following the previously published method (Lakra et al. 2015).

#### Electron microscopy

Young leaf sections (0.5–1 cm) were fixed first in a solution of 2% glutaraldehyde and then in 1% OsO_4_. Vacuum was applied to the dipped samples to ensure the penetration of fixative solution to the leaf pieces. Tissues were stained with uranyl acetate following dehydration in ethanol and embedded in Spurr’s medium. Samples were then sectioned and stained once more and observed under an electron microscope (JEOL) in Advanced Instrument Research Facility, Jawaharlal Nehru University. For ultrastructure analysis of leaf surface, the samples were prepared following the protocol of Zhou et al. (2013) and examined in an XL-30-ESEM (ZEISS) in Advanced Instrument Research Facility, Jawaharlal Nehru University.

#### Root anatomy study

The root of WT and *OsHBP1b* over-expressing rice seedlings were cut by free hand to get the transverse sections. The sections were stained with safranin (0.25% w/v) and methylene blue (0.25% w/v) for tissue demarcation and mounted in glycerine (10% v/v) and observed under light microscope (Leica) and photographs were taken with a digital camera fitted to the microscope.

#### Chlorophyll *a* fluorescence and photosynthesis related study

Chlorophyll *a* fluorescence and photosynthesis of the leaf of mature plants (third leaf from the top from 100 days old plants) was monitored at 25 °C undersupplied CO_2_ concentration (400 ppm), by using Infra-Red Gas Analyser (IRGA, LICOR-6400XT). Leaves were dark-adapted for 30 min prior to measurement of fluorescence. The minimal level of dark fluorescence (F_0_) was measured under weak modulated light and the maximal intensity of fluorescence (F_m_) was evoked by application of a short saturating light pulse (10, 000 μmol m^− 2^ s^− 1^). The maximal steady state photochemical efficiency (i.e. intrinsic quantum yield under dark adapted condition) was indicated by (F_v_ / F_m_), where F_v_ = F_m_ - F_0_. The electron transport rate (ETR) was estimated from the equation ETR = quantum yield * photosynthetic photon flux density (PPFD) * 0.84 * 0.5, where 0.84 represents the standard leaf absorptance and 0.5 denotes that the rate of PS-I photochemistry is in a match with PS-II. Photosynthesis of WT and *OsHBP1b* over-expressing plants was measured on a sunny day without dark adaptation.

#### SNPs and InDels analysis

The analysis of SNPs and InDels in IR64 and Pokkali was performed using the Rice SNP discovery database (http://14.139.61.3:8080/mjain/ricesnp/index.html) which holds the re-sequenced data for the rice cultivars (Jain et al. 2014).

#### Statistical analysis

Readings were obtained from a minimum of three biological replicates for each experiment. Data were analysed using ANOVA by GraphPad InStat3 software.

## Additional files

Additional file 1: **Figure S1.** Confirmation and molecular analysis of transgenic rice lines over-expressing *OsHBP1b*. (a) Tissue PCR analysis showed eight positive rice lines over-expressing *OsHBP1b*. Vector specific forward and gene specific reverse primer pair (F1R1) has been used here. (b) Southern blot analysis showed pattern of digestion (left) and copy number (right) of transgene in the *OsHBP1b* over-expressing plants. Two lines (L2 and L7) showed intense and clear band having a single copy of transgene and hence, further experimental analysis were done by using L2 and L7 lines. (c) Fold change in expression of *OsHBP1b* in *OsHBP1b* over-expressing plant as compared with WT, analyzed through quantitative real-time PCR. Expression of eEF was taken as reference and fold change was calculated using 2^-ΔΔCT^ method. (d) Western blot analysis showed higher OsHBP1b protein accumulation in the *OsHBP1b* over-expressing plants as compared with the WT. Error bars show standard error from triplicate experiments. ﻿Single asterisk show significant difference at *p* < 0.005 and double asterisk at *p* < 0.001. (TIF 7325 kb)

Additional file 2: **Figure S2.** Assessment of drought stress tolerance of *OsHBP1b* over-expressing rice seedlings through morphological, physiological and enzymatic analysis. (a) Photograph of WT and *OsHBP1b* over-expressing plants under drought conditions. Note the prominent difference in root morphology where *OsHBP1b* over-expressing plants have longer roots with more secondary branches as compared to the WT under drought stress. Bar diagrams showing (b) shoot and root length, (c) fresh weight, (d) Fv/Fm, (e) CAT activity, (f) APX activity and (g) SOD activity measured in the leaves of seedlings after 3 days of drought stress. The data represent means ± SE of three biological replicates. Single asterisk show significant difference at *p* < 0.005 and double asterisk at *p* < 0.001. (TIF 8735 kb)

Additional file 3: **Figure S3.** Assessment of high-temperature (45 °C) stress tolerance of *OsHBP1b* over-expressing rice seedlings through morphological, physiological and enzymatic analysis. (a) Photograph of WT and *OsHBP1b* over-expressing plants after 12 h of high-temperature (45 °C) conditions and subsequent recovery for 3 days. Note the prominent difference in root morphology where *OsHBP1b* over-expressing plants have longer roots with more secondary branches as compared to the WT under high temperature stress. Bar diagrams showing (b) shoot and root length, (c) fresh weight, (d) Fv/Fm, (e) CAT activity, (f) APX activity and (g) SOD activity measured in leaves of seedlings after 3 days of recovery. The data represent means ± SE of three biological replicates. Single asterisk show significant difference at *p* < 0.005 and double asterisk at *p* < 0.001. (TIF 8178 kb)

Additional file 4: **Figure S4.** Assessment of ABA responsiveness of *OsHBP1b* over-expressing rice seedlings through morphological and physiological analysis. (a) Photograph of WT and *OsHBP1b* over-expressing plants in response to exogenous ABA (5 uM). Note the prominent difference in root morphology where *OsHBP1b* over-expressing plants have longer roots with more secondary branches as compared to the WT in response to ABA. Bar diagrams showing (b) shoot and root length, (c) fresh weight, (d) Photograph of WT and *OsHBP1b* over-expressing plants in response to exogenous ABA (10 uM). Bar diagrams showing (e) shoot and root length, (f) fresh weight. The data represent means ± SE of three biological replicates. Single asterisk show significant difference at *p* < 0.005 and double asterisk at *p* < 0.001. (TIF 9550 kb)

Additional file 5: **Table S1.** List of primers used in this study and their sequences (5′ to 3′). (DOCX 16 kb)

Additional file 6: **Table S2.** SNPs and InDels analysis of *Saltol* QTL localized gene OsHBP1b (LOC_Os01g17260) in IR64 and Pokkali genotypes of *Oryza sativa* L. (DOCX 21 kb)

Additional file 7: **Table S3.** Segregation analysis of transgenic lines over-expressing OsHBP1b. (DOCX 15 kb)

## Data Availability

The data sets supporting this article are included in the article and in the additional files.
