# Supplementary figures and images for: A unique bZIP transcription factor imparting multiple stress tolerance in Rice
Source: Rice (N Y). 2019 Aug 2;12:58. doi: 10.1186/s12284-019-0316-8 (PMC6890918; doi:10.1186/s12284-019-0316-8)

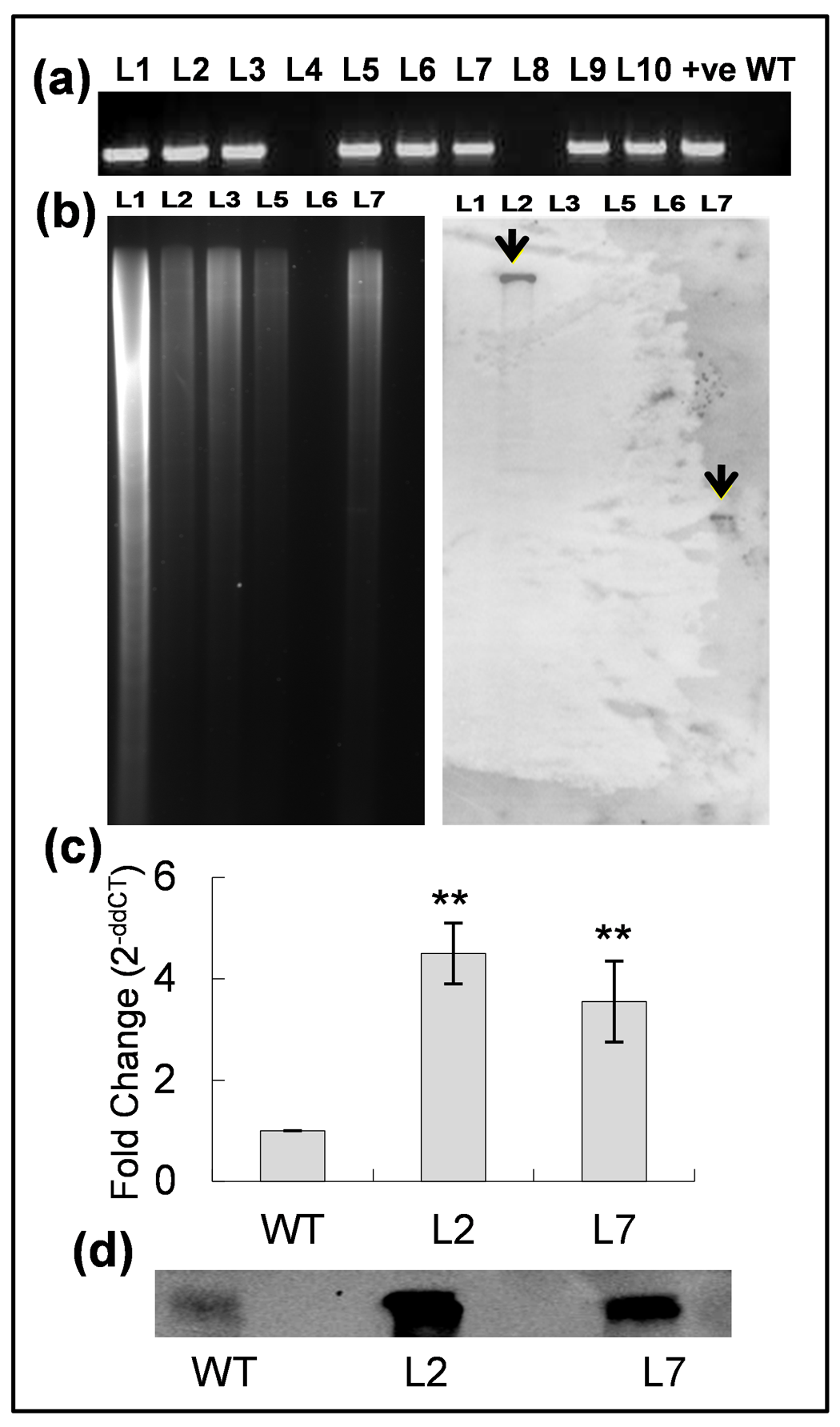

Supplement: Supplementary file 1 — Figure S1. Confirmation and molecular analysis of transgenic rice lines over-expressing OsHBP1b. (a) Tissue PCR analysis showed eight positive rice lines over-expressing OsHBP1b. Vector specific forward and gene specific reverse primer pair (F1R1) has been used here. (b) Southern blot analysis showed pattern of digestion (left) and copy number (right) of transgene in the OsHBP1b over-expressing plants. Two lines (L2 and L7) showed intense and clear band having a single copy of transgene and hence, further experimental analysis were done by using L2 and L7 lines. (c) Fold change in expression of OsHBP1b in OsHBP1b over-expressing plant as compared with WT, analyzed through quantitative real-time PCR. Expression of eEF was taken as reference and fold change was calculated using 2-ΔΔCT method. (d) Western blot analysis showed higher OsHBP1b protein accumulation in the OsHBP1b over-expressing plants as compared with the WT. Error bars show standard error from triplicate experiments. ﻿Single asterisk show significant difference at p < 0.005 and double asterisk at p < 0.001. (TIF 7325 kb) [file 12284_2019_316_MOESM1_ESM.tif]

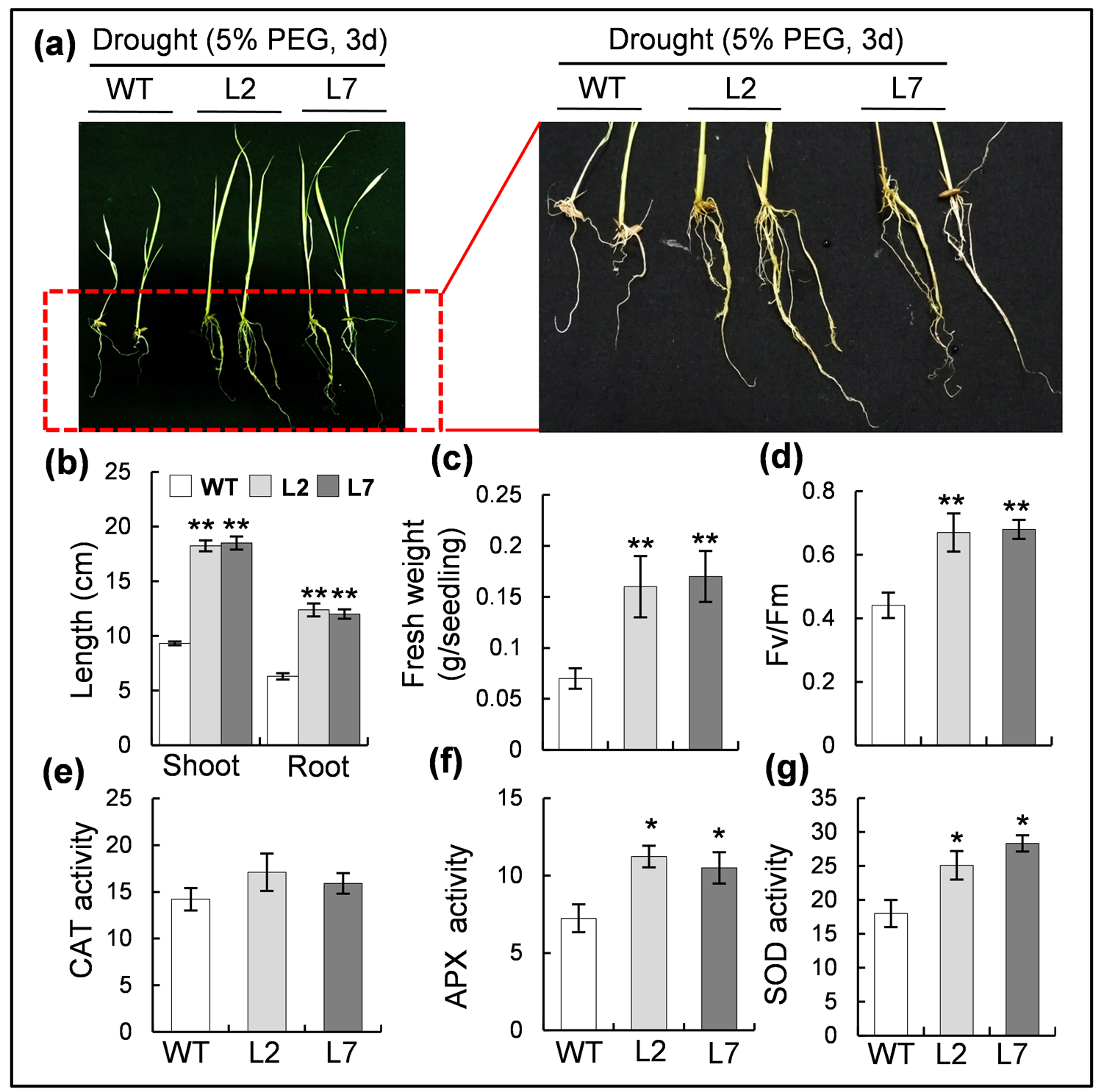

Supplement: Supplementary file 2 — Figure S2. Assessment of drought stress tolerance of OsHBP1b over-expressing rice seedlings through morphological, physiological and enzymatic analysis. (a) Photograph of WT and OsHBP1b over-expressing plants under drought conditions. Note the prominent difference in root morphology where OsHBP1b over-expressing plants have longer roots with more secondary branches as compared to the WT under drought stress. Bar diagrams showing (b) shoot and root length, (c) fresh weight, (d) Fv/Fm, (e) CAT activity, (f) APX activity and (g) SOD activity measured in the leaves of seedlings after 3 days of drought stress. The data represent means ± SE of three biological replicates. Single asterisk show significant difference at p < 0.005 and double asterisk at p < 0.001. (TIF 8735 kb) [file 12284_2019_316_MOESM2_ESM.tif]

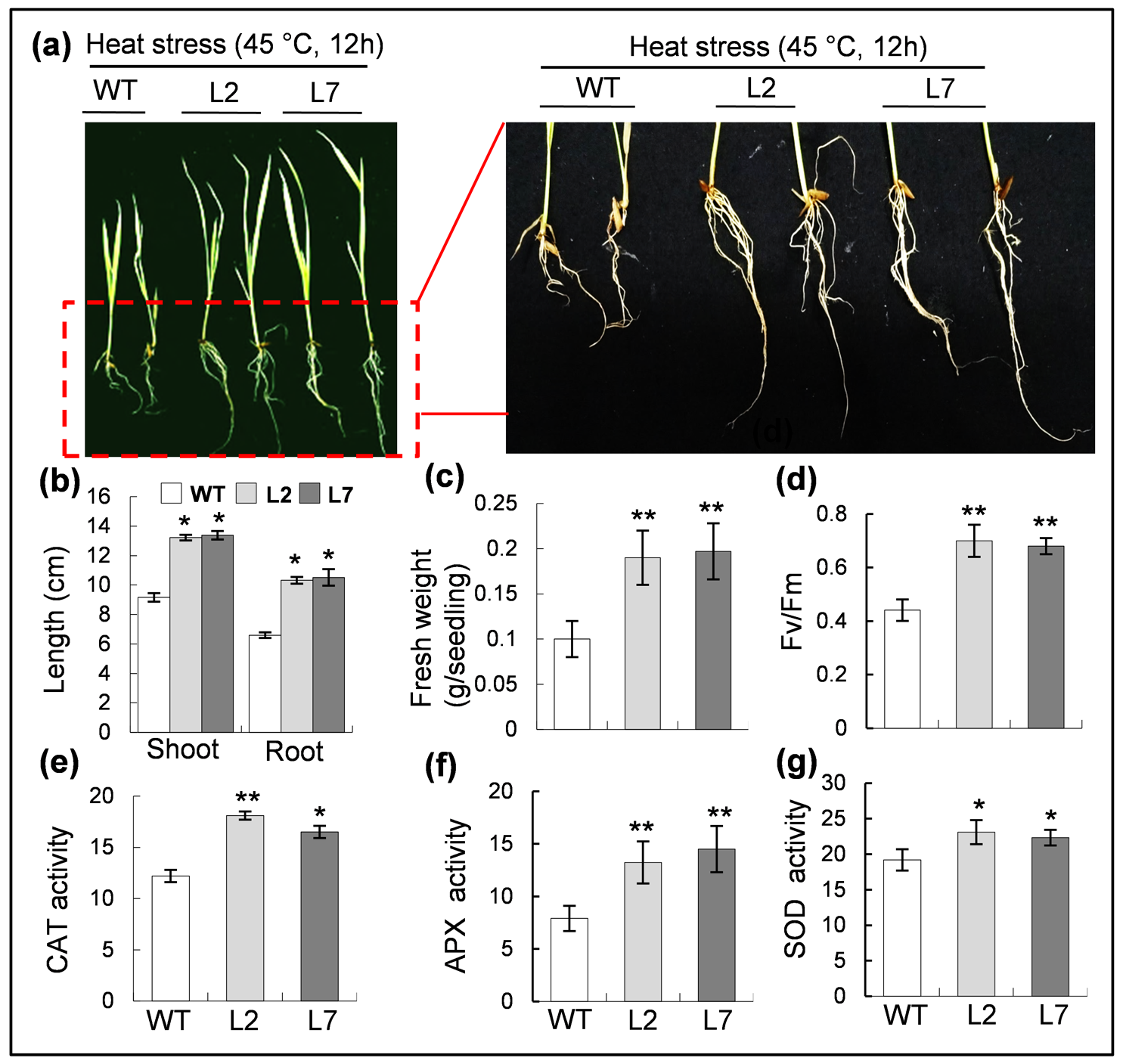

Supplement: Supplementary file 3 — Figure S3. Assessment of high-temperature (45 °C) stress tolerance of OsHBP1b over-expressing rice seedlings through morphological, physiological and enzymatic analysis. (a) Photograph of WT and OsHBP1b over-expressing plants after 12 h of high-temperature (45 °C) conditions and subsequent recovery for 3 days. Note the prominent difference in root morphology where OsHBP1b over-expressing plants have longer roots with more secondary branches as compared to the WT under high temperature stress. Bar diagrams showing (b) shoot and root length, (c) fresh weight, (d) Fv/Fm, (e) CAT activity, (f) APX activity and (g) SOD activity measured in leaves of seedlings after 3 days of recovery. The data represent means ± SE of three biological replicates. Single asterisk show significant difference at p < 0.005 and double asterisk at p < 0.001. (TIF 8178 kb) [file 12284_2019_316_MOESM3_ESM.tif]

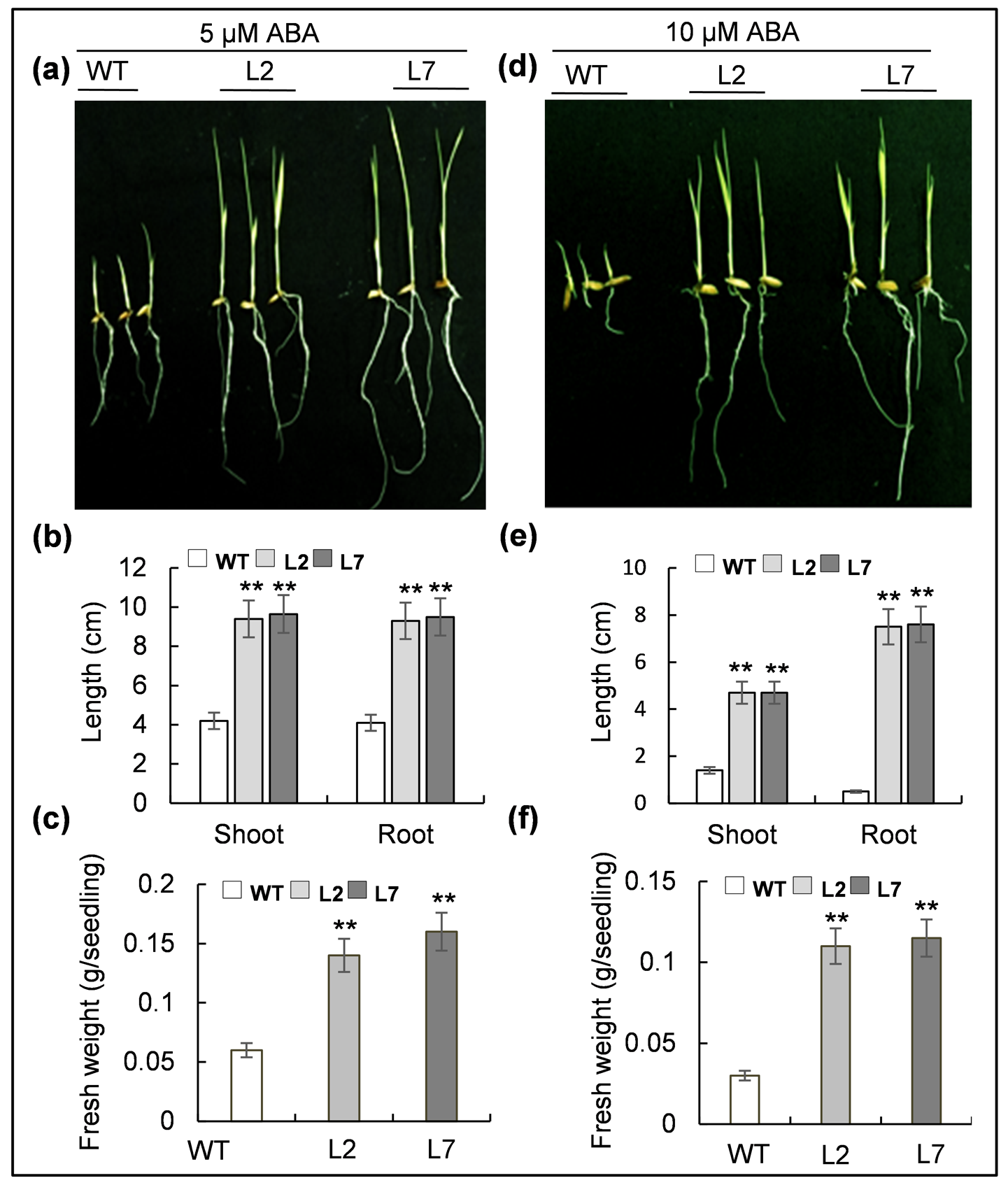

Supplement: Supplementary file 4 — Figure S4. Assessment of ABA responsiveness of OsHBP1b over-expressing rice seedlings through morphological and physiological analysis. (a) Photograph of WT and OsHBP1b over-expressing plants in response to exogenous ABA (5 uM). Note the prominent difference in root morphology where OsHBP1b over-expressing plants have longer roots with more secondary branches as compared to the WT in response to ABA. Bar diagrams showing (b) shoot and root length, (c) fresh weight, (d) Photograph of WT and OsHBP1b over-expressing plants in response to exogenous ABA (10 uM). Bar diagrams showing (e) shoot and root length, (f) fresh weight. The data represent means ± SE of three biological replicates. Single asterisk show significant difference at p < 0.005 and double asterisk at p < 0.001. (TIF 9550 kb) [file 12284_2019_316_MOESM4_ESM.tif]
