# Supplementary material for: A unique bZIP transcription factor imparting multiple stress tolerance in Rice
Source: Rice (N Y). 2019 Aug 2;12:58. doi: 10.1186/s12284-019-0316-8 (PMC6890918; doi:10.1186/s12284-019-0316-8)
Supplement: Supplementary file 5 — Table S1. List of primers used in this study and their sequences (5′ to 3′). (DOCX 16 kb) [file 12284_2019_316_MOESM5_ESM.docx]

| Gene | Forward Primer sequence (F) | Reverse Primer  sequence (R) |
| --- | --- | --- |
| OsHBP1b | GGAAGATCTGATGGCAGATATGAGCCC | GACTAGTCTATTCTTTCGGCCGAGCAAG |
| T-OsHBP1b | GGAAGATCTGATGGCAGATATGAGCCC | GACTAGTCTATTCTTTCGGCCGAGCAAG |
| qRT-OsHBP1b | GGATAGCCAACCTTCAGCAG | ATTTGGCCCATGTAGTTTGC |
| SOD (LOC_Os05g25850) | CCAGAAGCACCACGCCACCT | GATTGACATGGCCTCCGC |
| CAT  (LOC_Os06g51150) | ATGGATCCCTACAAGCATCGG | AGATGATAGTCCTCAAGGAGG |
| APX  (LOC_Os03g17690) | CGAGCCCATCAAGGAGGAGA | AGGTGGGGGTGCAGGTTTGTC |
| DREB1A  (LOC_Os04g46400) | CGCTGTCCACTTCCTCCTCC | TGCGCCGTGGGCGGCATTGGC |
| P5CS2  (LOC_Os05g38150) | GGGAGCAACTCACTGAAACTG | CAGTCCTGCTAAACTGTCATTA |
| NAC6  (LOC_Os03g60080) | ATGAGCGGCGGTCAGGACCT | TCTCGGCGATGATGGGGACGG |
| bZIP23  (LOC_Os01g31580) | ATGGGGTGCACGGCGTCGAAG | GGGAGAGCGCGGCGGCGGTG |
| HSTF  (LOC_Os03g06630) | CGAGTGGTGAACCCGGTGAAGGT | TACGTCTTGGTGAGGAACGGCGG |
| HSP90  (LOC_Os06g50300) | CCAGCTAAGAAGCTCCAAG | CTGAGGGTCTTCCTCGAG |
| RbcL  (LOC_Os10g21268) | CTTGGCAGCATTCCGAGTA | AAGAAGTAGGCCGTTGTCG |
| PsaA (LOC_Os10g38229 ) | CACGGTGTCTAAGGACACGTT | GACAGCGCCCATAAAGGTCTC |
| PsbA  (LOC_Os08g35420) | AGAGACGCGAAAGTACAAGC | AAGTTGCGGTCAATAAGGTA |
| LHCP2  (LOC_Os9g17740) | GAAGAAGATCAAGAACGGCCGA | TTGCCGGGGACGAAGTTGGT |
| CAO  (LOC_Os10g41780) | ATGACCACTGTGGCATCGCTGT | GATTCCTCCATATCTACCAAC |
| POR  (LOC_Os10g38229) | ATGGCTCTCCAAGTTCAGGC | ACGCTAAGGAACCCCGGCT |
| Actin | CAGCCACACTGTCCCCATCTA | AGCAAGGTCGAGACGAAGGA |

**Table S1:** **List of primers used in this study and their sequences (5′ to 3′)**
