# Supplementary material for: A unique bZIP transcription factor imparting multiple stress tolerance in Rice
Source: Rice (N Y). 2019 Aug 2;12:58. doi: 10.1186/s12284-019-0316-8 (PMC6890918; doi:10.1186/s12284-019-0316-8)
Supplement: Supplementary file 6 — Table S2. SNPs and InDels analysis of Saltol QTL localized gene OsHBP1b (LOC_Os01g17260) in IR64 and Pokkali genotypes of Oryza sativa L. (DOCX 21 kb) [file 12284_2019_316_MOESM6_ESM.docx]

**Table S2:** **SNPs and InDels analysis of *Saltol* QTL localized gene OsHBP1b (LOC_Os01g17260) in IR64 and Pokkali genotypes of *Oryza sativa* L.**

| Gene | Position | Reference (Nipponbare) | IR64 | Pokkali | Genomic feature |
| --- | --- | --- | --- | --- | --- |
| SNPs | 9928344 | G | G | A | Upstream |
|  | 9928439 | A | G | A | Upstream |
|  | 9928465 | C | T | C | Upstream |
|  | 9928525 | G | A | G | Upstream |
|  | 9928534 | G | A | G | Upstream |
|  | 9928757 | G | G | A | Upstream |
|  | 9930583 | T | C | T | Genic |
|  | 9930742 | G | T | G | Genic |
|  | 9930768 | C | T | C | Genic |
|  | 9931130 | G | G | A | Genic |
|  | 9931263 | C | C | A | Genic |
|  | 9931443 | G | G | A | Genic |
|  | 9931958 | C | C | A | Genic |
|  | 9931979 | C | C | T | Genic |
|  | 9932123 | G | A | G | Genic |
|  | 9932157 | C | C | G | Genic |
|  | 9932774 | A | G | A | Genic |
|  | 9933279 | G | G | A | Genic |
|  | 9933657 | G | G | T | Genic |
|  | 9933879 | T | G | T | Genic |
|  | 9934128 | C | T | C | Genic |
|  | 9935378 | A | G | A | Genic |
|  | 9935901 | A | A | T | Genic |
| InDels | 9928430 | T | TA | TAA | Upstream |
|  | 9930288 | GTA | GTA | G | Genic |
|  | 9932661 | T | T | TC | Genic |
