# Supplementary material for: A unique bZIP transcription factor imparting multiple stress tolerance in Rice
Source: Rice (N Y). 2019 Aug 2;12:58. doi: 10.1186/s12284-019-0316-8 (PMC6890918; doi:10.1186/s12284-019-0316-8)
Supplement: Supplementary file 7 — Table S3. Segregation analysis of transgenic lines over-expressing OsHBP1b. (DOCX 15 kb) [file 12284_2019_316_MOESM7_ESM.docx]

| Transgenic plant line | Total no. of tested seeds per plate | Resistant to hygromycin (R) | Susceptible to hygromycin (S) | Ratio (R:S) | Χ^2^ value |
| --- | --- | --- | --- | --- | --- |
| L2 | 60 | 44 | 16 | 2.8:1 | 0.07* |
| L7 | 60 | 45 | 15 | 3:1 | 0.03* |

**Table S3: Segregation analysis of transgenic lines over-expressing OsHBP1b**

All Χ^2^ value indicated significant fit the Mendelian segregation ratio 3:1 (p<0.05)
